# Mesenchymal stem cell therapy in hypertrophic and keloid scars

**DOI:** 10.1007/s00441-020-03361-z

**Published:** 2021-01-02

**Authors:** Christine Bojanic, Kendrick To, Adam Hatoum, Jessie Shea, K. T. Matthew Seah, Wasim Khan, Charles M. Malata

**Affiliations:** 1 Plastic & Reconstructive Surgery Department, Addenbrooke’s Hospital, Cambridge University Hospitals NHS Foundation Trust, Cambridge, UK; 2 Division of Trauma and Orthopaedics, Department of Surgery, Addenbrooke’s Hospital, University of Cambridge, Cambridge, UK; 3 School of Clinical Medicine, University of Cambridge, Cambridge, UK; 4 Cambridge Breast Unit, Addenbrooke’s Hospital, Cambridge University Hospitals NHS Foundation Trust, Cambridge, UK; 5 School of Medicine, Anglia Ruskin University, Cambridge & Chelmsford, UK

**Keywords:** Mesenchymal stem cells, Scar, Pain, Wound healing, Wound regeneration

## Abstract

Scars are the normal outcome of wound repair and involve a co-ordinated inflammatory and fibrotic process. When a scar does not resolve, uncontrolled chronic inflammation can persist and elicits excessive scarring that leads to a range of abnormal phenotypes such as hypertrophic and keloid scars. These pathologies result in significant impairment of quality of life over a long period of time. Existing treatment options are generally unsatisfactory, and there is mounting interest in innovative cell-based therapies. Despite the interest in mesenchymal stem cells (MSCs), there is yet to be a human clinical trial that investigates the potential of MSCs in treating abnormal scarring. A synthesis of existing evidence of animal studies may therefore provide insight into the barriers to human application. The aim of this PRISMA systematic review was to evaluate the effectiveness of MSC transplantation in the treatment of hypertrophic and keloid scars in in vivo models. A total of 11 case-control studies were identified that treated a total of 156 subjects with MSCs or MSC-conditioned media. Ten studies assessed hypertrophic scars, and one looked at keloid scars. All studies evaluated scars in terms of macroscopic and histological appearances and most incorporated immunohistochemistry. The included studies all found improvements in the above outcomes with MSC or MSC-conditioned media without complications. The studies reviewed support a role for MSC therapy in treating scars that needs further exploration. The transferability of these findings to humans is limited by factors such as the reliability and validity of the disease model, the need to identify the optimal MSC cell source, and the outcome measures employed.

## Introduction

Wounds to the skin are caused by mechanical, thermal, and chemical trauma. Scars (or cicatrix) are the normal outcome of wound repair and involve a co-ordinated inflammatory and fibrotic process. Eventually, the scars remodel and become soft, flat, pale, and unobtrusive. When a scar does not resolve, persistent chronic inflammation can cause excessive scarring that lead to a range of abnormal phenotypes which clinically manifest as hypertrophic and keloid scars.

Hypertrophic scars affect nearly one in five people who suffer from burns and the risk of scarring increases with the time taken to heal (Chipp et al. 2017). They can also occur following incisional closure, a standard part of surgical procedures. Typically appearing within 2 months of injury, the disease process can be protracted and therefore carries significant societal and financial cost over a long period of time (Gangemi et al. 2008). Keloid scars impact tens of millions of people worldwide, and there is strong evidence of a significant genetic predisposition (Bayat et al. 2003; Santos-Cortez et al. 2017). In contrast to hypertrophic scars, keloid scars can appear much later post-injury and are characterised by extension beyond the original area of the trauma. Ultimately, hypertrophic and keloid scars result in significant impairment of quality of life (Bock et al. 2006). In addition to cosmetic consequences, these abnormal scars can have functional implications including restricted mobility, pain, and pruritus (Bijlard et al. 2017; Lee et al. 2004).

Excess scarring may persist and often recurs after multiple interventions (Darzi et al. 1992; Gauglitz et al. 2011). Most patients suffer from neuropathic pain and pruritus, and the mainstay of treatment is conservative therapy (Argirova et al. 2006). However, existing treatment options are generally unsatisfactory for patients and doctors alike. In particular, surgery, which is mainly focused on scar excision, has a very high recurrence rate whether used alone or in combination with depot steroids (Berman et al. 2009; Furtado et al. 2012; Wilson 2013). Strategies aimed at scar growth suppression include topical treatments such as retinoic acid, imiquimod, and corticosteroid injections (Jacob et al. 2003; Janssen De Limpens 1980). These remedies tend to demonstrate only short-term efficacy (Berman et al. 2009; Cação et al. 2009). Repeated steroid injections are nevertheless efficacious. Pressure therapy and silicone gel cream or sheets stand out as clinically useful and widely used measures both therapeutically and preventatively (Ai et al. 2017; Kim et al. 2014). Modalities such as radiotherapy, cryotherapy, and lasers have either high failure rates, and/ or carry risk of adverse events, not to mention high cost (Manuskiatti and Fitzpatrick 2002; Puri and Talwar 2009; Song et al. 2014; Steinstraesser et al. 2011). Therefore, there is mounting interest in innovative methods to treat hypertrophic and keloid scars. Emerging studies have therefore taken a different approach and focussed on cell-based therapies such as mesenchymal stem cells (MSCs) (Fung et al. 2017).

MSCs are adult multipotent stromal cells that can be readily harvested from various sites such as bone marrow, adipose, and umbilical tissue (Baksh et al. 2007; Khan et al. 2008). MSCs can be expanded ex vivo and cultured under specific conditions to promote particular cellular effects. Due to their low immunogenicity, MSCs are frequently transplanted allogeneically for the treatment of inflammatory conditions (Kabat et al. 2020). MSCs exert their anti-inflammatory and anti-fibrotic paracrine effects via the chemokines and microvesicles that they secrete (Badiavas et al. 2003; Horwitz and Dominici 2008; Rani et al. 2015). Excessive scarring involves undesired inflammation that results in deposition of immature extracellular matrix (ECM) by fibroblasts and myofibroblasts (Barallobre-Barreiro et al. 2019). Whilst tissue native MSCs play a key role in potentiating this process, there is evidence to suggest that transplanted MSCs are instead able to attenuate inflammation and promote a return to homeostasis (Chen et al. 2009; Ren et al. 2008). MSCs may achieve this by mediating macrophage class switch from a proinflammatory M1 to anti-inflammatory M2 phenotype (Cho et al. 2014). MSCs also have the potential to negatively modulate ECM deposition, possibly via promoting a T-cell response that results in the downregulation of TGF-β1, a key regulator of collagen synthesis (Huang et al. 2015; Spiekman et al. 2014).

Despite the interest in MSCs, there is yet to be a human clinical trial that investigates the potential of MSCs in treating excessive scarring. A synthesis of existing evidence of animal studies will therefore provide insight into the barriers to human application. The aim of this systematic review was to evaluate the effectiveness of MSC transplantation in the treatment of hypertrophic and keloid scars in in vivo models.

## Materials and methods

A literature search was performed using PubMed, Web of Science, and Cochrane Database from conception to May 2020. The following search terms were used: ((((((((MSC) OR Mesenchymal Stem Cell) OR Mesenchymal Stromal Cell) OR Multipotent Stem Cell) OR Multipotent Stromal Cell) OR Stem Cell)) AND ((Keloid) OR Hypertrophic)) AND Scar.

**Fig. 1:**
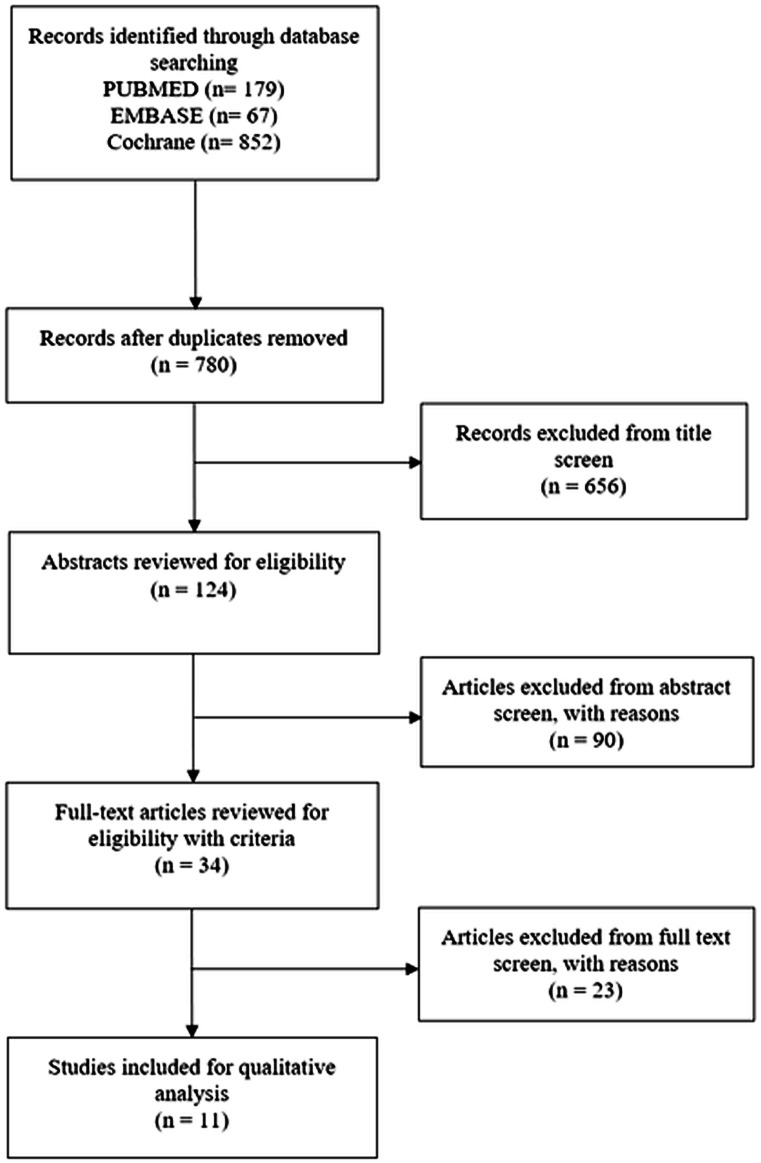
Flow diagram of search strategy

**Fig. 2:**
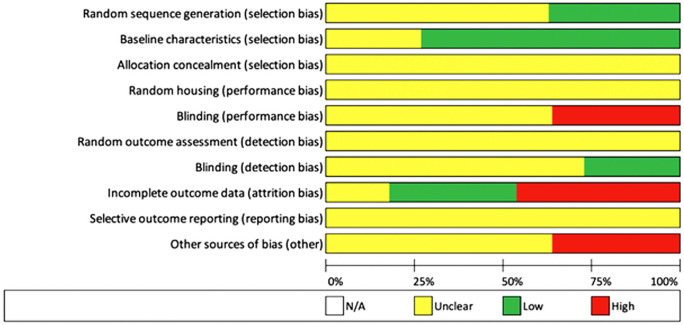
Overall risk of bias

We adhered to the Preferred Reporting Items for Systematic Reviews and Meta-Analyses (PRISMA) guidelines and included case control, cohort studies, case series, and randomised controlled trials (Moher et al. 2009). A total of 1098 studies were subjected to the inclusion/exclusion criteria, yielding a final 11 studies for qualitative analysis (Fig. 1). Studies that evaluated MSC or MSC-conditioned media transplantation as therapies were included. Studies that assessed in vivo models were included. Studies of all design were included. Literature reviews, systematic reviews, and case reports were excluded but were reverse-reference searched to maximise yield. Studies with only in vitro experiments were excluded. All included studies were published in the English language, and all unpublished, inaccessible, and retracted literature were excluded. CB and KT carried out the search independently. Risk of bias was assessed by AH and JS using the SYRCLE RoB tool (Table 1; Fig. 2) (Hooijmans et al. 2014).

**Table 1:** Summary of level of bias of individual studies

Study	Random sequence generation	Baseline characteristics	Allocation concealment	Random housing	Blinding (performance)	Random outcome assessment)	Blinding (detection)	Incomplete outcome data	Selective outcome reporting	Other sources of bias	Overall risk of bias
Yates et al. (2017)	Some	Some	Some	Some	Some	Some	Low	Some	Low	Some	Some
Liu et al. (2018)	Low	Low	Some	Some	High	Some	Some	Some	Low	Some	High
Hu et al. (2019)	Some	Low	Some	Some	High	Some	Low	Low	Low	High	High
Domergue et al. (2016)	Some	Low	Some	Some	Some	Some	Some	Low	Low	Some	Some
Liu et al. (2014)	Some	Low	Some	Some	Some	Some	Some	High	Low	Some	High
Yates et al. (2017)	Some	Low	Some	Some	Some	Some	Low	High	Low	Some	High
Liu et al. (2014)	Low	Low	Some	Some	Some	Some	Some	High	Low	Some	High
Hu et al. (2020)	Some	Some	Some	Some	Some	Some	Some	High	Low	High	High
Li et al. (2016)	Low	Some	Some	Some	Some	Some	Some	Low	Low	Some	Some
Zhang et al. (2015)	Some	Low	Some	Some	High	Some	Some	Low	Low	High	High
Foubert et al. (2017)	Some	Low	Some	Some	High	Some	Some	High	Low	High	High

## Results

A total of 11 studies were identified (Tables 2, 3, and 4) (Domergue et al. 2016; Foubert et al. 2017; Hu et al. 2019, 2020; Li et al. 2016; Liu et al. 2018, 2014; Yates et al. 2017; Yates et al. 2017; Zhang et al. 2015). A total of 156 subjects were treated with MSCs or MSC-conditioned media. There were no significant complications reported in any of the studies. Ten studies assessed the effectiveness of MSCs or MSC-conditioned media in treating hypertrophic scars and one in keloid scars. All studies were case control studies.

**Table 2:** MSC isolation and characterisation

Author	MSC source	Method of tissue extraction	MSC characterisation	MSC treatment
Hu et al. (2019)	Murine bone marrow	Needle aspiration from tibia and femur	Flow cytometry (CD150+/CD74+), tri-lineage differentiation (osteogenic, adipogenic, chondrogenic)	Passages 8–13 harvested at 70%
Hu et al. (2019)	Murine bone marrow	Needle aspiration from tibia and femur	Flow cytometry (CD105 + /CD73+), tri-lineage differentiation (osteogenic, adipogenic, hepatogenic)	Passages 8–13 harvested at 70%
Liu et al. (2018)	Human adipose	Surgical excision of redundant tissue from surgical operations	Flow cytometry (CD105+/CD90+/CD34-/CD45-/CD19-), adipogenic and osteogenic differentiation	Passages 3–4
Foubert et al. (2017)	Porcine adipose	Surgical excision of inguinal fat pad	Flow cytometry (CD90+/CD45-)	Untreated, re-suspended in buffer solution and delivered two hours following isolation
Yates et al. (2017)	Human bone marrow	Immortalised cell line	Flow cytometry (CD105+/CD14-/CD34-/CD45-), tri-lineage differentiation (osteogenic, adipogenic, chondrogenic)	Passage 4 harvested at 70%
Yates et al. (2017)	Human bone marrow	Needle aspiration from posterior iliac crest	Flow cytometry (CD105+/CD14-/CD34-/CD45-), tri-lineage differentiation (osteogenic, adipogenic, chondrogenic)	Passage 3 harvested at 70%
Domergue et al. (2016)	Human adipose	Dermolipectomy	Flow cytometry (CD73+/CD90+/CD34-/CD14-)	End of passage 1
Li et al. (2016)	Human adipose	Liposuction	Flow cytometry (CD73+/CD90+/CD34-/CD14-), adipogenic and osteogenic differentiation	Passages 3–5 starved for 24 h at 80–90% confluence prior to supernatant collection
Zhang et al. (2015)	Rabbit adipose	Surgical excision of inguinal fat pad	Flow cytometry (CD73+/CD90+/CD34-/CD14-), adipogenic and osteogenic differentiation	Passage 3 harvested at 80–90%
Liu et al. (2014)	Human bone marrow and rabbit bone marrow	Human: bone marrow biopsy Rabbit: bone marrow needle aspiration	Flow cytometry (CD105+/CD90+/CD34-/CD45-), chondrogenic and osteogenic differentiation	Passage 3–4
Liu et al. (2014)	Rabbit bone marrow	Needle aspiration from tibia and femur	Flow cytometry (CD105 + /CD90 + /CD34-/CD45-), adipogenic and osteogenic differentiation	Passage 4–6

**Table 3:** Characteristics of studies observing MSC effects on scar formation from wounds

Author	Method of delivery	Model	Scar characteristics	Subject (*n* =)	Control (*n* =)	Follow-up duration	Assessment	Outcome
Hu et al. (2019)	1 × 10^6^ cells/ml (200 μl of 2 × 10^5^ MSC) in conditioned medium (CM), bone marrow concentrate (BMC) CM, or BMC-treated MSC CM or control (Dulbecco’s modified Eagle medium (DMEM)) injected subcutaneously into each wound on day 14, 21, 28	Rabbit	Hypertrophic scar (HS)-Full-thickness, 1 cm diameter circular wound, ear	MSC CM (*n* = 4) BMC CM (*n* = 4) BMC-treated MSC CM (*n* = 4)	DMEM into contralateral ear wound (*n* = 12)	Day 35	Macroscopic appearance, histology and immunohistochemistry, collagen gel contraction assay	Improved wound appearance and reduced HS formation in BMC-treated MSC CM compared with other groups. BMC-treated MSC CM also reduced fibroblasts and HS contracture
Foubert et al. (2017)	0.25 × 10^6^ cells sprayed topically onto each square centimetre of wound or lactated Ringer's (LR)	Pig	HS-Full-thickness 2-mm depth, 58-cm^2^ wound, flanks	MSC (*n* = 12)	LR sprayed topically onto contralateral flank wound (*n* = 12)	Day 60 or 180	Macroscopic appearance, histology, biomechanical assessment of elasticity, collagen deposition assay, digital planimetry	Reduced scarring, improved scar pigmentation and epidermal remodelling. Reduced collagen deposition and enhanced elastic fibre length compared with control groups. Reduced scar tissue hardness and vascularisation. Higher levels of IL-6 and TNF-alpha compared with control. No difference in wound contracture
Yates et al. (2017)	2 × 10^7^ cells/ml (50 μl of 1 × 10^6^ MSCs), MSC-tenacin C (TNC), MSC-fibroblast co-culture mixture or MSC-TNC fibroblast co-culture mixture subcutaneously injected into each wound	Murine	HS-Full-thickness, 8 mm punch wounds, dorsum	MSC-TNC on WT mice (*n* = 3) MSC-TNC on Chemokine receptor 3 (CXCR3) -/- mice (*n* = 3) MSC-TNC fibroblast on WT mice (*n* = 3) MSC-TNC fibroblast on CXCR3-/- mice (*n* = 3)	HA (*n* = 3) MSC-HA (*n* = 3) on both wild type and CXCR3-/- mice	Day 30	Macroscopic appearance, histology, immunohistochemistry, immune cell infiltration analysis and fluorescent apoptosis assay (caspase-3 staining)	Reduced scarring in MSC-TNC fibroblast co-culture mixture group compared with other groups. Reduced fibroblast apoptosis when co-cultured with MSCs
Yates et al. (2017)	Wounds filled with tenascin C in a collagen/GAG-based (TPolymer) or MSC-TPolymer or left untreated and covered with Tegaderm	Murine	HS-Full-thickness, 6-mm punch wound, dorsum	MSC-TPolymer on wild type (WT) mice (*n* = 3) MSC-polymer on CXCR3-/- mice (*n* = 3) TPolymer on WT mice (*n* = 3) TPolymer on CXCR3-/- mice (*n* = 3)	No treatment on contralateral dorsal wound on both wild type and CXCR3-/- mice (*n* = 12)	Day 3, 7, 14, 21, 60 or 90	Macroscopic appearance, histology, immunohistochemistry	Reduced scarring by improved collagen alignment in MSC-TPolymer group compared with TPolymer group only. Improved wound repair and dermal maturation in MSC-TPolymer group. TPolymer enhances MSC survival
Li et al. (2016)	1000 μl of varying concentrations of MSC CM derived from passage 3–5 MSCs at 80–90% confluence subcutaneously injected into each wound	Murine	HS-Full-thickness, 1-cm^2^ area wound, dorsum	10% MSC CM (*n* = 6) 20% MSC CM (*n* = 6) 40% MSC CM (*n* = 6) 80% MSC CM (*n* = 6)	DMEM (*n* = 6)	Day 14	Macroscopic appearance, histology, immunohistochemistry	Reduced scar formation, reduced skin fibrosis, faster wound healing in MSC CM group. Decreased collagen deposition, reduced collagen I and III expression in a concentration-dependent manner
Zhang et al. (2015)	200ul of MSC or MSC CM or DMEM injected into centre of each wound	Rabbit	HS-Full-thickness, 1-cm^2^ area wound, ear	MSC (*n* = 4) MSC CM (*n* = 4)	Untreated (*n* = 4) DMEM on contralateral ear wound of each group (*n* = 12)	Day 35	Macroscopic appearance, histology, immunohistochemistry, ultrasonography to assess scar thickness	Reduced scar hypertrophy in both MSC and MSC CM group. MSC group more effective than MSC CM group. Reduced scar tissue height
Liu et al. (2014)	6.25 × 10^6^ cells/ml (80 μl of 5 × 10^5^ MSCs) or PBS injected intradermally circumferentially around each wound	Rabbit	HS-Full-thickness, 7-mm punch wound, ear	Human MSC (*n* = 6) rabbit MSC (*n* = 6) human MSC-small interfering RNA (*n* = 6) human MSC-H_2_O_2_ (apoptosis model) (*n* = 6) human MSC-with capsase-3 inhibitor (anti-apoptotic model) (*n* = 6)	PBS (*n* = 6)	Day 14 or 28	Macroscopic appearance, histology, immunofluorescence, digital planimetry, TUNEL (Terminal deoxynucleotidyl transferase dUTP nick end labelling) staining for detecting apoptosis	Attenuated HS formation and reduced HS height in human MSC group. MSC groups showed significant MSC apoptosis shortly after transplantation. Therapeutic effect was attenuated in the anti-apoptotic model
Liu et al. (2014)	1000 μl of MSC (1 × 10^5^ cells) or PBS injected intra-arterially via ear artery	Rabbit	HS-Full-thickness, 9-mm round wound, ear	MSC transduced with p53 shRNA (*n* = 4) MSC transduced with control short hairpin RNA (shRNA) (*n* = 4)	PBS (*n* = 4)	Day 21, 28 or 35	Macroscopic appearance, histology, immunohistochemistry	Prevented HS formation in a p53 mediated manner. Knockdown of p53 in MSC increased HS fibroblast proliferation

**Table 4:** Characteristics of studies observing MSC effects on formed scars

Author	Method of delivery	Model	Scar characteristics	Subject (*n* =)	Control (*n* =)	Follow-up duration	Assessment	Outcome
Domergue et al. (2016)	100 μl of MSC (1 × 10^5^ cells) or stromal vascular fraction (SVF) (1 × 10^6^ MSCs) injected subcutaneously into four points of grafted human scar samples	Murine	HS-Full-thickness, 2-cm^2^ human xenograft on dorsum	MSC (* n* = 10) SVF (* n* = 10)	Phosphate buffer solution (PBS) (*n* = 10)	Day 49 or 63	Macroscopic appearance, histology, immunohistochemistry, collagen content assay	Reduced scar hypertrophy and improved fibrosis remodelling in both MSC and SVF group. MSC group was more effective. Reduced scar thickness and collagen content in treated groups
Liu et al. (2018)	200 μl of MSC CM or DMEM injected subcutaneously into four sites within keloid xenograft	Murine	Keloid scar-Full-thickness, 1 cm^2^ human xenograft on dorsum	MSC CM (*n* = 4)	DMEM (*n* = 4) Untreated (*n* = 4)	Day 28	Macroscopic appearance, histology, immunohistochemistry, Bromodeoxyuridine / 5-bromo-2′-deoxyuridine (BrdU) cell proliferation enzyme-linked immunosorbent assay (ELISA), phosphatidylserine apoptosis assay	Increased keloid shrinkage and reduced CD31 and CD68 staining. Reduced keloid fibroblast proliferation. No change in apoptosis of KS fibroblast in treated group
Hu et al. (2019)	200 μl of MSC CM, MSC CM+ Botox, or DMEM injected subcutaneously into each wound on days 7, 14, and 21	Murine	HS-Full-thickness, 6 mm^3^ xenograft on dorsum	MSC CM (*n* = 4) MSC CM + Botox (* n* = 4)	DMEM (*n* = 3) Botox (* n* = 4)	Day 28	Macroscopic appearance, histology, immunofluorescence, collagen deposition assay, fibroblast apoptosis assay (caspase-7 staining)	Reduced scarring in MSC CM+ Botox group. Most marked scar weight reduction in MSC CM+ Botox group. Decreased collagen deposition and increased fibroblast apoptosis in MSC CM+ Botox group

### MSC isolation and characterisation

Six studies used bone marrow MSCs: two of murine origin (Hu et al. 2019, 2020), two of human origin (Yates et al. 2017; Yates et al. 2017), one of rabbit origin (Liu et al. 2014), and one study included both human and rabbit origin MSCs (Liu et al. 2014). Five of the studies that employed bone marrow MSCs harvested cells by needle aspiration from either the tibia, femur or posterior iliac crest (Hu et al. 2019, 2020; Liu et al. 2014; Liu et al. 2014; Yates et al. 2017). One study, by Yates et al*.* (Yates et al. 2017) used bone marrow MSCs derived from an immortalised cell line. Five studies utilised adipose MSCs: three of human (Domergue et al. 2016; Li et al. 2016; Liu et al. 2018), one of porcine (Foubert et al. 2017) and one of rabbit origin (Zhang et al. 2015). Three studies experimented MSCs from inguinal fat pad or redundant tissue from surgical operations (Foubert et al. 2017; Liu et al. 2018; Zhang et al. 2015). Domergue et al. (2016) extracted MSCs by dermolipectomy and Li et al. (2016) by liposuction. Whilst all the studies applied flow cytometry to characterise MSCs, only four satisfied the International Society for Cellular Therapy (ISCT) criteria for defining MSCs by also performing tri-lineage differentiation (Dominici et al. 2006; Hu et al. 2019, 2020; Yates et al. 2017; Yates et al. 2017). Five studies performed bi-lineage differentiation only (Li et al. 2016; Liu et al. 2018, 2014; Liu S. et al. 2014; Zhang et al. 2015).

### MSC treatment and delivery

Most studies passaged MSCs at least three times. Only two studies used MSCs from earlier passage; Domergue et al. (2016) used passage one, and Foubert et al. (2017) did not passage the cells at all. Interestingly, two studies harvested MSCs beyond the eighth passage (Hu et al. 2019, 2020). Studies transplanted varying concentrations of MSCs but at similar volumes of around 200 μl. Two studies administered 1000 μl (Li et al. 2016; Liu et al. 2014), whilst three studies dispensed less than 100 μl of MSCs (Domergue et al. 2016; Liu. et al. 2018; Yates et al. 2017). Yates et al*.* (Yates et al. 2017) did not specify the quantity given. The routes of MSC administration were highly variable. Eight of the eleven studies delivered MSCs or MSC-conditioned media by subcutaneous injection. Of these, four studies specified further; two injected four points of the wound (Domergue et al. 2016; Liu et al. 2018), one injected into the centre of each wound (Zhang et al. 2015), and the fourth delivered MSCs by circumferential intradermal injection into each wound (Liu et al. 2014). Of the remaining three studies, one study delivered MSCs onto the wound via an aerosol (Foubert et al. 2017), one applied the MSCs to fill the wound defect (Yates et al. 2017), and one injected MSCs intra-arterially (Liu et al. 2014). Five of the eleven studies utilised MSC-conditioned media (Hu et al. 2019, 2020; Li et al. 2016; Liu et al. 2018; Zhang et al. 2015). Two studies used chemokine receptor 3 (CXCR3) knockout mice, which are known to scar excessively when wounded (Yates et al. 2017; Yates et al. 2017). Four studies employed internal controls by injecting MSCs on the contralateral side of the animal subject (Foubert et al. 2017; Hu et al. 2019; Yates et al. 2017; Zhang et al. 2015). Five studies utilised Dulbecco’s modified Eagle media (DMEM) (Hu et al. 2019, 2020; Li et al. 2016; Liu et al. 2018; Zhang et al. 2015), and three applied phosphate buffer solution (PBS) as controls (Domergue et al. 2016; Liu et al. 2014; Liu et al. 2014). Other control groups comprised lactated Ringer’s solution (LR), hyaluronic acid (HA), and no treatment as a control (Foubert et al. 2017; Yates et al. 2017; Yates et al. 2017). The majority of studies followed up wound progression for at least 28 days.

### Disease model

Eight studies evaluated the effectiveness of MSCs in preventing hypertrophic scar formation (Table 3), and three studies examined MSC therapy on formed scars. In the latter, one study assessed keloid scars and included four subjects. Six studies assessed murine, four used rabbit, and one utilised a porcine subject. All the induced wounds were full dermal-thickness but varied in size and location, with the majority being circular punch wounds inflicted on the dorsum of murine subjects. Four studies inflicted full-thickness punch wounds on the ears of rabbit subjects (Hu et al. 2019; Liu et al. 2014; Liu et al. 2014; Zhang et al. 2015). Three studies (Table 4) created full-thickness skin wounds on human skin samples which were then xenografted onto murine subjects (Domergue et al. 2016; Hu et al. 2020; Liu et al. 2018).

### Treatment outcomes and complications

All studies assessed wounds in terms of macroscopic appearance and histology with most including immunohistochemistry. No complications were reported by any of the studies. Gross appearance was evaluated in all studies using high-resolution photography, and all studies reported positive improvements in various measured parameters in the MSC-treated group compared with controls. Eight studies described reduced scar hypertrophy in the MSC-treated group compared with controls (Domergue et al. 2016; Foubert et al. 2017; Hu et al. 2019, 2020; Li et al. 2016; Yates et al. 2017; Yates et al. 2017; Zhang et al. 2015). Two studies reported that MSC-treated subjects attenuated hypertrophic scar formation (Liu et al. 2014; Liu et al. 2014). One study evaluated keloid size and found greater scar shrinkage following treatment (Liu et al. 2018). Several studies assessed collagen characteristics using assays of collagen gel contraction (Hu et al. 2019), collagen deposition (Foubert et al. 2017; Hu et al. 2020), and collagen content (Domergue et al. 2016). All studies reported reduced collagen deposition and reduced collagen contracture in the MSC-treated group compared with controls. Two studies assessed fibroblast apoptosis. Hu et al. (2020) found increased fibroblast apoptosis by staining for caspase-7. Liu et al. (2018) measured the presence of phosphatidylserine in the outer layer of the phospholipid bilayer as a surrogate marker of apoptosis and found no change in the MSC-treated group compared with control. Another study used TUNEL (terminal deoxynucleotidyl transferase dUTP nick end labelling) staining to assess MSC apoptosis and found that a significant proportion of MSCs underwent apoptosis after administration onto a wound (Liu et al. 2014). Three of the eleven studies assessed scar thickness, with two using digital planimetry (Foubert et al. 2017; Liu et al. 2014) and one using ultrasonography (Zhang et al. 2015). All studies reported reduced scar tissue height and hardness. Yates et al. (2017), by staining caspase-3 with a fluorescent probe, found reduced caspase-3, suggesting improved fibroblast survival following MSC co-transplantation.

## Discussion

Although the outcomes reported in this review generally favour MSC transplantation in treating excessive scarring and did not report complications, it is difficult to draw reliable conclusions due to the heterogeneity of the studies. This arises from various aspects; there was significant variability in the cell source, cell treatment, method of delivery, and the disease model used to assess efficacy. Most studies demonstrated moderate to high overall risk of bias as they were aiming to different and more specific questions relevant to MSC use. Nevertheless, this systematic review provides a useful summary and helps inform future study design.

The properties of MSCs can vary according to the cell source. Consistent with the existing literature, most of our studies examined adipose MSCs (AMSCs) and bone marrow MSCs (BMMSCs) (Kabat et al. 2020). Both of these cell sources have their relative advantages for use in treating scars. AMSCs offer a greater capacity to proliferate ex vivo compared with other cell sources (Peng et al. 2008) and therefore may be suitable for large scale off-the-shelf preparations at greater cost-effectiveness. They may also be more abundant, less invasive to harvest, and are often available as medical waste in many cosmetic surgery procedures. BMMSCs may represent a less heterogenous cell population (Liu et al. 2013) but exhibit senescence at earlier passage (Burrow et al. 2017). An important consideration is that the anti-inflammatory properties of MSCs could differ by cell source. Particular studies suggest that AMSCs may be superior in promoting an M1 to M2 phenotype transition in macrophages that favour resolution of inflammation (Heo et al. 2019). This is relevant as macrophages are a key mediator of the pathogenic process of excessive scarring (Feng et al. 2019; Hesketh et al. 2017). In addition, certain MSCs demonstrate a greater ability to engraft onto lesions and can therefore produce more sustained effects (Burk et al. 2016). One study in this review compared human and rabbit cell sources and found both cell sources to be equally efficacious (Liu et al. 2014). Harvesting MSCs from animals rather than humans may be more convenient but the immunogenic consequences of xenogeneic transplantation with human recipients are yet to be thoroughly investigated. Although heterogeneity of MSC origin and culture condition among the included studies may affect the reliability of conclusions drawn from them, it is reassuring that positive effects were observed across multiple cell sources. This indicates that MSCs regardless of origin have the potential to treat hypertrophic and keloid scars. Future studies should aim to identify the best cell source for treating excessive scarring.

Significant heterogeneity was also observed between the studies in terms of culture conditions and treatment delivery methods. The literature suggests that pre-conditioning MSCs with inflammatory cytokines may serve to promote an anti-inflammatory MSC phenotype (Saldaña et al. 2019). Similarly, following co-culture with fibroblasts, a cell type prevalent in inflamed scars, MSCs express greater levels of anti-inflammatory cytokines (Suzuki et al. 2017). This suggests that treating MSCs in conditions reflective of the scar environment might potentiate their effectiveness when used in transplantation. Conversely, serum-free culture conditions appear to enhance the anti-fibrotic properties of MSCs in vivo (Yoshida et al. 2018). This may represent a potential challenge as the optimal culture protocol should promote an anti-fibrotic response without compromising the anti-inflammatory properties of MSCs. One way of circumventing this could be to stimulate MSCs under a particular set of culture conditions, and then harvesting the conditioned media that contains bioactive extracellular vesicles (EVs). The MSCs can then be resuspended and grown under a different set of culture conditions to promote secretion of different bioactive substances. Indeed, several studies in our review showed that a cell-free treatment using MSC-conditioned media can be effective (Hu et al. 2019, 2020; Li et al. 2016; Zhang et al. 2015).

On the other hand, it is difficult to identify the best MSC delivery method. MSCs injected into the circulation appear to engraft well into wounds (Deng et al. 2005), but carry a risk of interacting with cytokines and drugs present in the serum, which may alter MSC function (Javorkova et al. 2018). In contrast, MSCs injected directly into a lesion of interest could delocalise rapidly (Burk et al. 2016) and therefore still have the potential to exert off-site effects (Devine et al. 2003). Although there were no complications reported in any of the studies in this review, several factors have the potential to influence MSC biodistribution and therefore clinical efficacy following administration. It has been reported that pulmonary complications relating to IV administration of MSCs could be dependent on the cell suspension formulation (Deak et al. 2010). Other studies suggest that following initial localisation in the lungs following systemic administration, MSCs can home to areas of inflammation (Rustad and Gurtner 2012). Although useful in cases of isolated skin pathology, undesired offsite effects may be observed in cases of other underlying systemic inflammation (Gholamrezanezhad et al. 2011). There is also evidence to show that the migration and proliferation of MSCs at skin wounds can be a function of MSC expression of adhesion molecules including junction adhesion molecule A (JAM-A) (Wu et al. 2015). Likewise, chemokines such as CCR7 also appear to promote MSC migration to skin wounds (Sasaki et al. 2008). For the purposes of treating scars, it appears that local administration may be preferable, with recent studies demonstrating safety in animals via subcutaneous (Tappenbeck et al. 2019) and topical (Beyazyildiz et al. 2014) routes. Robust experiments that compare methods of MSC delivery in treating scars should address this ambiguity.

It remains uncertain whether interpretations drawn from animal models of excessive scarring can be transferred directly to inform treatment in humans. Most of the studies in this review assessed the effects of MSCs on the degree of hypertrophy during the scarring process. This probably does not replicate the human disease where patients typically present with a fully formed scar. Nevertheless, it may inform whether MSCs can be implemented at the time of injury (in high risk patients) or shortly after or in conjunction with surgical scar treatment as a means of preventing primary or recurrent hypertrophic or keloid scars. Genetic models of hypertrophic scarring may confer high reproducibility. There are existing gain-of-function models such as the Tight Skin 2 mouse which exhibit increased fibrosis following injury (Long et al. 2014), presumably due to increased collagen III alpha-1 expression (Long et al. 2015). Instead of a gain-of-function model, the two studies by Yates et al*.* (Yates et al. 2017; Yates et al. 2017) captured in this review utilised a previously validated knockdown model by targeting the CXCR3 gene (Yates et al. 2010). Whilst both methods may be informative for in vivo studies of hypertrophic scarring, they do not reflect the pattern of genetic predisposition in humans (Zhu et al. 2013), and the knock-down target does not correlate with known protective genetic variants (Sood et al. 2015). It is suggested that concomitantly xenografting human skin cells into the wound may improve the validity of the mouse burns model by promoting a more extensive scar phenotype (Ibrahim et al. 2014; Momtazi et al. 2013). However, this could be confounded by the immunogenic effects of xenografting skin onto an immunocompetent mouse (Racki et al. 2010). Nevertheless, the studies in this review that conducted xenografting of human skin into mouse defects did not observe graft rejection (Domergue et al. 2016; Hu et al. 2019; Liu et al. 2018).

Another issue relates to the time-course of scar pathogenesis. Most mouse models develop mature hypertrophic or keloid scarring within days to weeks after burn injury and weeks to months after incisional injury (Kim et al. 2018), unlike the longer time course of human disease. In humans, excessive scarring can occur after months (Gangemi et al. 2008), with biomolecular evidence of active disease at up to a year later (Van Der Veer et al. 2011). There is evidence in the literature to support the potential use of the Red Duroc porcine model, which develops scarring over months instead, and therefore better recapitulates the human process (Harunari et al. 2006; Zhu et al. 2003, 2004). We captured one study by Foubert et al. (2017) that was able to utilise this model in order to undertake an extended follow-up period of six months, when active scar growth was still observed. Whilst all of the studies demonstrated sustained benefit and did not report recurrence up to the end point of follow-up, keloid and hypertrophic scars are known in humans to recur after many months to years following successful treatment (Furtado et al. 2012). Therefore, the short lifespan of murine models may not permit sufficient longitude to assess whether the benefits of MSC therapy is sustained. Future studies of porcine models with long follow-up periods may facilitate this.

In order to fully exploit the beneficial effects of MSCs in treating scars, it is important to establish a dose-response relationship. The studies in this review varied significantly in the amount of MSC or MSC-conditioned media used, but all reported positive outcomes. Only one study examined the effects of varying the dose of MSC-conditioned media used and found a dose-response relationship (Li et al. 2016). It is unclear whether the same relationship may be observed in treatment with MSCs of varying concentration and there is evidence in models of ischaemic injury that higher doses of MSCs do not always confer greater therapeutic benefit (Yavagal et al. 2014). Therefore, a relevant future study might aim to determine the maximum tolerated dose (MTD) for MSCs in treating keloid and hypertrophic scars. The method of delivery might influence this, as appropriate dosage for intravenous injection may be derived from the weight of the subject, whereas intralesional delivery may require the volume of the scar of interest to be calculated. Digital planimetry, as employed by several studies here, may be a viable method of achieving this (Foubert et al. 2017; Liu et al. 2014). Ascertaining the MTD will also inform safe dosages that do not evoke adverse effects (Karussis et al. 2010). Six studies in this review treated scars with MSCs, four studies used conditioned media, and one compared the two. There has been an emerging body of evidence to support the use of conditioned media, which contains bioactive extracellular vesicles (EVs) that may be the active therapeutic ingredient of MSCs (Furuta et al. 2016). As a cell-free therapy, it is possible that EVs are less immunogenic and may therefore be more suitable for large-scale production from allogeneic sources (Monguió-Tortajada et al. 2017).

Outcome measures utilised by in vivo studies can limit their transferability to humans. Whilst reduction in scar size and improvement in histological appearance may reflect the cosmetic benefits of treatment, it is unclear how it affects scar symptoms. As pain and pruritis are the main symptoms of hypertrophic and keloid scars (Lee et al. 2004), functional assessments in animals may be crucial before undertaking human trials. For example, there are well-validated and quantifiable behavioural measures such as vocalisation that reflect pain in mice (Kurejova et al. 2010). Assessing the degree of physical activity such as time spent digging (Shepherd et al. 2018) could potentially reveal the functional implications of contractures resulting from scars, although this could be dependent on the position of the lesion. Aside from looking to reduce the amount of scarring, there is a range of symptoms that can be caused by excessive scarring, and so separate studies may be required to evaluate the differential benefits of MSC therapy and to determine a personalised approach according to the specific symptom.

## Conclusion

The present review suggests that mesenchymal stem cell (MSC) therapy can be an effective method of treating hypertrophic and keloid scars across a range of cell sources and animal models and does not cause significant complications. However, there is inadequate high-level evidence of in-human studies to support clinical efficacy in humans. There are several areas that need to be addressed before proceeding to human trials. This includes the identification of a reliable, reproducible, and validated animal model, and a standardised method of MSC delivery to allow a dose-response relationship to be established. The similar positive results observed to date with MSCs and MSC-conditioned media are encouraging and should be explored further by assessing the efficacy of MSC-derived extracellular vesicles, as this will carry significant implications for cost-effectiveness in treating humans at a population scale.
